# Setting an Optimal *α* That Minimizes Errors in Null Hypothesis Significance Tests

**DOI:** 10.1371/journal.pone.0032734

**Published:** 2012-02-28

**Authors:** Joseph F. Mudge, Leanne F. Baker, Christopher B. Edge, Jeff E. Houlahan

**Affiliations:** Department of Biology, University of New Brunswick, Saint John, New Brunswick, Canada; Genentech, United States of America

## Abstract

Null hypothesis significance testing has been under attack in recent years, partly owing to the arbitrary nature of setting *α* (the decision-making threshold and probability of Type I error) at a constant value, usually 0.05. If the goal of null hypothesis testing is to present conclusions in which we have the highest possible confidence, then the only logical decision-making threshold is the value that minimizes the probability (or occasionally, cost) of making errors. Setting *α* to minimize the combination of Type I and Type II error at a critical effect size can easily be accomplished for traditional statistical tests by calculating the *α* associated with the minimum average of *α* and *β* at the critical effect size. This technique also has the flexibility to incorporate prior probabilities of null and alternate hypotheses and/or relative costs of Type I and Type II errors, if known. Using an optimal *α* results in stronger scientific inferences because it estimates and minimizes both Type I errors and relevant Type II errors for a test. It also results in greater transparency concerning assumptions about relevant effect size(s) and the relative costs of Type I and II errors. By contrast, the use of *α* = 0.05 results in arbitrary decisions about what effect sizes will likely be considered significant, if real, and results in arbitrary amounts of Type II error for meaningful potential effect sizes. We cannot identify a rationale for continuing to arbitrarily use *α* = 0.05 for null hypothesis significance tests in any field, when it is possible to determine an optimal *α*.

## Introduction

A well-known problem associated with null hypothesis significance tests (NHST) is the arbitrariness of the chosen experimental significance level, alpha (*α*). Yet the practice of comparing an observed *p*-value to an arbitrary *α*, usually *α* = 0.05, remains widespread as “methodological orthodoxy” in science [1], [2]. There is large body of literature that advocates for the abandonment of NHST [1]–[5], and although we recognize that NHST is frequently misused, we do not wish to contribute to the bashing that is unlikely to garner much attention or end such a deeply entrenched practice among scientists. Instead, we posit that “*abuses non toll it sum*”, that abuse does not preclude proper use. The ease with which a correctly-interpreted null hypothesis significance test can be used as a decision-making tool causes it to continue to be favoured in most scientific fields. The goal of these tests should be to provide us with conclusions in which we have the highest possible confidence. Thus, the logical decision-making significance threshold, *α*, should be the value that minimizes the probability, or occasionally, the cost of making any relevant error. In the former case, this would make the goal of the statistical test to avoid making an erroneous conclusion, while in the latter it would make the goal of the statistical test to avoid making a costly erroneous conclusion. We feel that doing statistics for purposes other than these would be outside the realms of pure and applied science.

### The Case for a New Approach to Setting α

**Figure 1:**
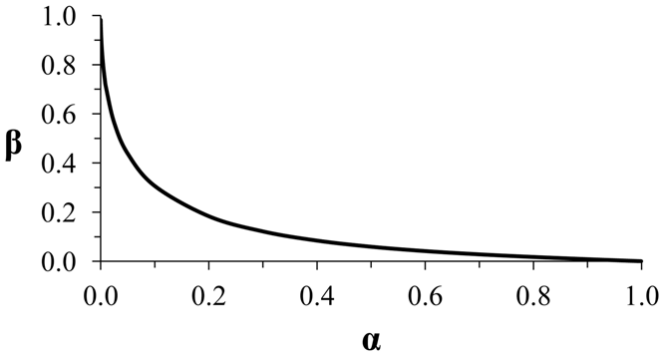
The non-linear relationship between *α* and *β*. The relationship between *α* and *β* for an independent 2-sample, 2-tailed *t*-test with n_1_ = n_2_ = 10, and critical effect size = 1 *σ*.

In traditional NHST there are two types of errors, rejecting the null hypothesis when it is true (Type I error) and failing to reject the null when the alternate hypothesis is true (Type II error). Alpha is set to address the Type I error rate – it is the probability of making a Type I error that we are willing to accept in a particular experiment. The choice of an *α* level will determine the probability of a Type II error (*β*) for a study with a given sample size and critical effect size. Decreasing the probability of Type I error increases the probability of Type II error and vice-versa (Figure 1). Because *α* determines the power (1 - *β*) to detect effects of specified sizes, then using the standard *α* = 0.05 makes implicit decisions about the effect sizes a researcher will be likely to consider significant, if they exist. The standard *α* = 0.05 also arbitrarily determines the chance of Type II error relative to Type I error for meaningful potential effect sizes. These implicit decisions associated with using *α* = 0.05 are often both unrealized and unrealistic. Appropriate conclusions from statistical tests should involve explicit considerations about the magnitude of effect that would be important to be able to detect, if it were real, and whether the probabilities of Type I and Type II error reflect the relative seriousness of the consequences of a Type I vs. Type II error. Considering critical effect sizes and the relative consequences of Type I and Type II errors should not be perceived as an unnecessary extra step of statistical testing that can be avoided, because decisions about these factors are unwittingly made using the traditional approach of setting *α* = 0.05. For a given study, it will not be possible to detect some effect sizes at *α* = 0.05, and when the researcher simply fails to consider how setting *α* = 0.05 can affect *β* for a meaningful potential effect size, they are unknowingly providing a study-specific cost ratio that may be heavily weighted in favour of reducing Type I errors, at the expense of experimental power, or *vice versa*, depending on the sample size of the study. Turning a blind eye to Type II errors, in favour of controlling a Type I error rate, allows *β* to fluctuate as a function of the sample size and variability. Although the traditional approach of ignoring Type II error probabilities may be easy, it can result in poor decisions. Our goal is to improve upon an obviously flawed hypothesis-testing system while working under the constraints of that system, because we acknowledge that researchers are not very likely to abandon an approach that is so easy to use and so widely understood.

Current practice involves a bias against accepting a falsehood (Type 1 error), where rejecting a truth (Type II error) is regarded as “healthy scepticism” [6], implying that there is much lower cost associated with Type II errors relative to the cost associated with Type I error [7]. We set *α* at a value other than zero because, while we want to limit our probability of falsely concluding there is an effect (Type I error), we also want to limit the probability of missing a real effect (Type II error). If one does not care at all about missing real effects (Type II errors), then a *β* level of 1 becomes acceptable and Type I error can be minimized by simply setting *α* = 0. The acceptability or magnitude of the potential for Type II errors should be an area of concern for all researchers, no matter the type of study performed. There are many cases where we believe that failing to detect a real effect, should be considered at least as serious, if not more serious than falsely detecting a non-existent effect. Should natural resource industries be encouraged to design low power environmental impact studies that would never have any chance of detecting anything but extreme environmental impacts using *α* = 0.05? In this case, a Type I error would result in loss of economic opportunity, while Type II error would result in environmental damage and associated economic costs [8]. We feel that, despite the difficulty in its quantification, the ratio of the importance of Type I vs. Type II errors should always be discussed, rather than disregarded.

Arguments for using a single, arbitrary value of *α* for all studies include: i) that it provides a consistent approach to statistical analysis and; ii) that it an objective approach that avoids value judgements; and iii) that it is easy to use. Arguments i) and ii) are incorrect because *p*-values are dependent on sample size. Setting *α* = 0.05 is not a truly consistent approach among studies because as sample size increases, the observed effect size required to produce a significant result using *α* = 0.05 decreases, so studies with different sample sizes require different observed effect sizes to yield a significant result. Because of this, consistently using *α* = 0.05 is not an objective approach. Subjectivity is merely shifted away from the choice of *α* to the choice of sample size, such that if a researcher wants to find statistical significance using *α* = 0.05 they should conduct a test with a large sample size (while small sample sizes could be used in the same way for cases in which the desired result is non-significance). While argument iii), the ease of use of *α* = 0.05 is certainly a benefit of using an arbitrary *α*, it is not necessarily good science. Given the absence of a strong rationale for continuing to use a single, arbitrary value for *α* among studies, what should be the basis for setting *α* in individual studies?

We argue that, in almost all contexts, the goal of statistical testing is to aid us in making conclusions that limit the probabilities of making mistakes, whether they be Type I or II errors. We think a strong case can be made that in most studies (and perhaps all) *α* should be set with the objective of either minimizing the combined probabilities of making Type I or Type II errors at a critical effect size, or minimizing the overall cost associated with Type I and Type II errors given their respective probabilities. This can be done rather simply if researchers explicitly consider the relative importance of avoiding Type I vs. II errors and estimate, *a priori*, what would constitute a relevant critical effect size. This makes it possible to set a study-specific optimal *α* level that can minimize either the average of the probabilities of Type I and Type II error at the critical effect size, or instead minimize the cost-weighted average of Type I and Type II errors. The optimal *α* approach can prevent the inflated overall error rates (combined probability of either a Type I or Type II error) that result from arbitrarily using *α* = 0.05, and requires that the user be transparent concerning detectability of *a priori* critical effect sizes and the relative importance of avoiding Type I and II errors. We describe a straightforward and flexible method for setting study-specific optimal *α* levels that is easily applicable in all pure and applied areas of scientific research and leads to optimal, evidence-based conclusions. We propose an approach to setting an optimal *α* level that has the flexibility to minimize either the combined probabilities of Type I errors and relevant Type II errors, or the minimize the relative cost of errors, and can incorporate specified or unknown critical effect sizes, relative costs of Type I and Type II errors or prior probabilities of hypotheses. This simple method can be used to derive an optimal *α* level that can be used by any researcher, setting NHST on a course of scientifically valid use and in a manner that leads to better decisions.

The optimal *α* level for any null hypothesis significance test depends on i) the prior probabilities that the null and alternate hypotheses are true, which are typically unknown but can therefore be assumed to be equal under Laplace's principle of indifference [9], ii) the relative costs of Type I and II errors, which are also often unknown but are assumed equal when the goal is to minimize combined probabilities of Type I and Type II error, iii) the critical effect size, iv) the sample size of the study, and v) the variability in the data. The remainder of this paper describes how the optimal *α* approach deals with prior probabilities, costs of Type I and II errors, and critical effect size estimation, provides a list of steps required to calculate an optimal *α* for any NHST. Case studies of the optimal *α* approach applied to previously published data [10]–[12] are also provided.

### Optimal α – the basic approach

The combined probability of making a Type I error or a relevant Type II error for a particular study (*ω*) is the average of *α* and *β* if we assume that the prior probabilities of the alternate and the null hypotheses to be equal (Equation 1).
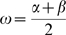
(1)In the absence of reliable information concerning prior probabilities (as is often the case in science), the assumption of equal prior probabilities is the only rational assumption [9]. However, if the probabilities of the alternate and null hypotheses are known and unequal, the probabilities of Type I and Type II error should still be averaged as in Equation 1, but with *α* and *β* each multiplied by their relative prior probabilities of the null and alternate hypotheses being true, respectively.

**Figure 2:**
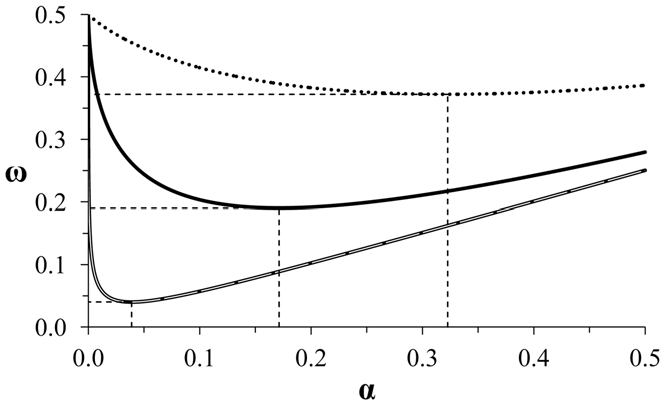
Determination of optimal *α* from the *a priori* combined probabilities of Type I and Type II error. *α* and *ω* (the average of Type I and Type II error) for independent, 2-tailed, 2-sample *t*-tests (n_1_ = n_2_). Data are for 3 (dotted line), 10 (solid line), and 30 (double line) samples per group, with critical effect sizes of 1 SD of either group. Drop lines indicate the minimum average of Type I and Type II error and its associated value of *α*.

Due to the nonlinear but negative and monotonic nature of the relationship between *α* and *β* (Figure 1), it is possible, through iterative examination of *ω* over a range of *α* values, to identify a unique combination of *α* and *β* that minimizes the combined probability of Type I and Type II error (*ω*) for a desired critical effect size, in a study with a given sample size (Figure 2).

### Incorporating unequal costs of Type I and Type II errors

**Figure 3:**
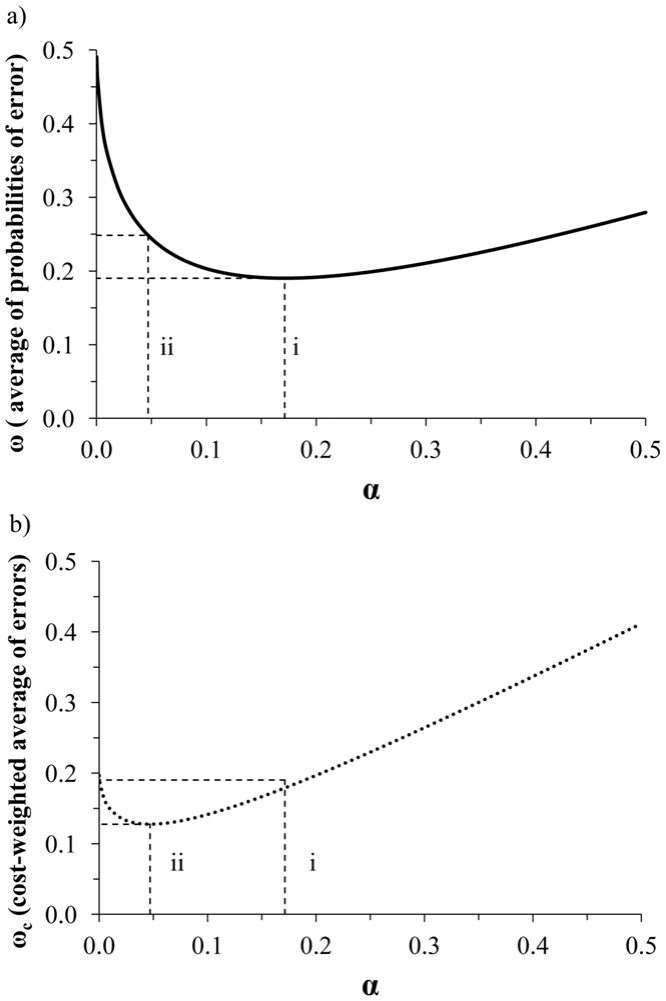
The average of the probabilities of Type I and Type II error, ω (a) and the cost-weighted probability of errors, ω_c_ (b). The combined probabilities of Type I and Type II error, *ω* (a), and the cost-weighted probability of errors, *ω*_c_ (b). The *α* level at i) minimizes average error (assuming a Type I/Type II error cost ratio of 1), while the *α* level at ii) minimizes the cost-weighted probability of errors at a Type I/Type II error cost ratio of 4.

Although minimizing the combined probabilities of Type I and Type II errors is likely the main goal of statistical testing for situations of pure scientific inquiry, research in the applied sciences may be more interested in statistical testing for the purpose of minimizing the overall costs of error (Figure 3). Overall costs of error can be minimized by choosing the *α* level associated with the minimum average of *α* and *β* weighted by the relative costs of Type I and Type II errors, *ω*_c_ (Equation 2).
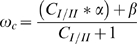
(2)Where C_I/II_ is the relative cost of Type I/Type II errors. In some cases it may be clear that one type of error is more serious than the other, either in terms of financial cost (e.g. when looking at the effects of a particular level of catch on an economically important fish population), human health (e.g. when screening for side effects of cancer treatment drugs), or research progress (e.g. any ‘basic’ science question). In these cases, optimal *α* level should be set to minimize the probability-weighted average of the relative costs of Type I and Type II errors (or equivalently, minimizing the cost-weighted average of *α* and *β*) instead of minimizing the combined probabilities Type I and Type II error. Setting decision-making thresholds in a way that accounts for the relative costs of Type I and Type II errors is an area of enquiry that has been largely unexplored in many scientific fields and it is particularly relevant to research where the costs of Type I and II errors are seen as potentially ‘estimable’ (e.g. environmental effects monitoring, pharmaceutical testing, or disease treatment efficacy). Minimizing overall cost of error implies that while making conclusions based on evidence is still a priority, some of the overall confidence in the conclusion should be sacrificed to make it more likely that when errors do occur, they are the least costly type of error (see Field *et al.* [13] for a discussion of this technique for environmental monitoring and management). If there are known financial costs associated with Type I and Type II errors, then calculating the relative cost of Type I/Type II error is simple (divide the cost of Type I error by the cost of Type II error), but we acknowledge that financial costs associated with one or both types of error are often unknown. In some cases it maybe be possible for consensus about the relative seriousness (i.e. cost) of Type I/Type II errors to be reached among stakeholders, and where dollar values cannot be used to quantify relative cost of error (e.g. an agreement that the consequences of Type I error are twice as serious as the consequences of Type II error). Methods for quantifying the relative costs of Type I/Type II error will therefore be study specific and will not be discussed further here, although we hope this work will generate more exploration of the relative costs of error in scientific research. Our opinion is that unless there is a strong justification for unequal costs of Type I and Type II errors, statistical testing in science should remain unbiased by costs of error and Type I and Type II errors should be treated as equally serious, allowing for the minimization of combined probabilities of Type I and Type II error.

### Determining a critical effect size

A critical effect size is the magnitude of effect in the response variable that would be considered important to detect if it exists, and can be determined prior to conducting an NHST. All studies ought to include some discussion of what size of an effect would constitute a meaningful difference from the null hypothesis [14]. It has been argued that researchers should be far more interested in the size of the effect observed than in its statistical significance [15] and we would agree that effect sizes are often not given enough attention in science. There is a vast body of literature that argues for the use of an effect size statistic [16], and ∼60 different effect sizes measures have been described [17]. Calculating *β* requires an explicit decision about the ‘critical’ effect size that a researcher would want to be able to detect, if real, and this must be decided upon, *a priori*. We do not advocate a researcher use the observed effect size in a study to determine the optimal *α* and *β*, this is acknowledged as a misleading, and completely confounded use of power analysis [18]. Many researchers have, and will continue to, struggle with setting a critical effect size because in basic science (and often in applied science) there is no completely objective rationale for choosing a critical effect size, and there is often no consensus among researchers concerning what magnitude of effect would be considered meaningful. Nonetheless, most researchers do have opinions about what effect sizes would be too small to be considered significant and what effect sizes would be too large to be considered non-significant. Devising a study-specific critical effect size and incorporating it into the calculation of *α* enables researchers to be transparent about these opinions and lowers the probability that the choice of *α* does not result in relevant effect sizes leading to ‘non-significant’ conclusions and irrelevant effect sizes leading to ‘significant’ conclusions. If there is no clear rationale for a specific effect size, multiple critical effect sizes could be used and results could be presented for ‘small’, ‘intermediate’ and ‘large’ critical effect sizes.

### Sample size and variability

This paper describes how to determine the optimal *α* after data have been collected, such that the sample size and estimated population variability are inherent and immutable characteristics of the experimental design. Contrary to the case with critical effect size, we think that it is most appropriate to use the observed variability and sample size of your data set, in order to yield the best estimate of power. This would be an appropriate and informative use of a power analysis; using the achieved sample size, *a priori* critical effect size and estimated variability, in order to most accurately determine Type II error probability. The optimal *α* can also play an important role in experimental design if used before data are collected (i.e. to determine the minimum sample size required to achieve a desired overall probability of error), in much the same way that one would use a prospective power analysis to design an experiment.

## Results and Discussion

To provide examples of how the optimal *α* approach can be applied for actual scientific research, we decided to re-analyse the results of two simple, typical null hypothesis significance tests from high-profile journals and one null hypothesis significance test used as an example in a statistics textbook. These examples will show how the optimal *α* approach affects data interpretation for different types of statistical tests in different fields, under circumstances of high and low statistical power.

### Case study 1: t-test with low power

First, consider a study comparing mountain yellow-legged frog (*Rana muscosa*) tadpole densities between naturally fishless lakes and lakes that had undergone complete removal of previously introduced rainbow trout (*Oncorynchus mykiss*) and brook trout (*Salvelinus fontinalis*) three years before sampling the tadpole population [10]. One of the study objectives was to test whether the effects of previously introduced fish on amphibian abundance were still present 3 years after the removal of the introduced species. The author tested the hypotheses, *H*_O_: there is no difference in mean number of tadpoles/10 m of shoreline between naturally fishless and fish removal lakes (*μ*_1_ = *μ*_2_), and *H*_A_: there is a difference in mean number of tadpoles/10 m of shoreline between naturally fishless and fish removal lakes (*μ*_1_≠*μ*_2_). The number of tadpoles/10 m of shoreline was measured in 8 naturally fishless lakes and 3 lakes that had undergone removal of previously introduced fish (i.e. n_1_ = 8 and n_2_ = 3). The mean numbers of tadpoles/10 m of shoreline observed were 29.62 and 10.1 at fishless control lakes and fish removal lakes, respectively, with an observed pooled standard deviation of 17.27 tadpoles/10 m of shoreline. The author performed an independent, 2-sample, 2-tailed *t*-test on these data, reported a *p*-value of 0.14, and considered the result non-significant, presumably using the standard *α* of 0.05 (although their choice of *α* was not explicitly stated).

The conclusion of the *t*-test depends on the method used to choose *α* and the desired critical effect size (Table 1). As there was no justification for unequal prior probabilities of hypotheses or costs of Type I and Type II errors apparent in the introduction of the paper, we assumed equal prior probabilities and costs of errors. In the absence of a strong rationale for any particular critical effect size, we chose to calculate optimal *α* levels for 3 different potential critical effect sizes; an effect size we consider to be ‘large’, representing a difference equal to 1.5 *σ* or a ±90% difference from the control mean, and 2 smaller effect sizes representing differences equal to 1*σ* and 0.5*σ* or ±60% and ±30% difference from the control mean, respectively. Using the traditional *α* of 0.05, the averages of Type I and Type II error at the critical effect size were 0.272, 0.394, and 0.474, for the large, medium and small effect sizes, respectively. Using the ‘optimal *α*’ approach, the averages of Type I and Type II errors were smaller, at 0.202, 0.319, and 0.443, for the large, medium and small effect sizes, respectively. The associated optimal *α* levels were 0.191, 0.266, and 0.323, so using optimal *α* has increased the probability of making a Type I error in all cases but dramatically lowered the probability of making Type II errors. In fact, for *α* = 0.05, using a small or medium critical effect size resulted in a >75% probability of failing to detect a true difference. Even when the critical effect size was ±90% difference from the control mean there was almost a 50% chance of missing a real effect of that size. This is a special concern when the major conclusion is one of ‘no effect’ as was the case in this paper. Using the optimal *α* method, we would conclude that the experimental design was not appropriate for detecting a small effect size – the minimum chance of making a mistake using optimal *α* is 44.3%, which is not substantially different than a coin toss (note: the probability of making a mistake using *α* = 0.05 was 47.4%). However, the minimum probability of making an error was 31.9% for a medium effect size and 20.2% for a large effect size and in both cases using the optimal *α* approach would have resulted in reaching the opposite conclusion (i.e. there was a significant difference in tadpole density between fishless lakes and fish-removed lakes). So, if the objective is to minimize the probability of making an error (Type I or II) – and there is rarely, if ever, a different and rational objective – then the author made the wrong conclusion regardless of his choice of effect size, and should have concluded that there was an insufficient number of samples to be able to make any strong conclusion, but that there was better evidence to for a difference between lake types than for no difference between lake types.

**Table 1:** Probabilities of Type I (*α*), Type II (*β*) and average error (*ω*), with corresponding test conclusions for large, medium and small effect sizes (*δ*) using standard *α* levels and by setting *α* to minimize combined probabilities of Type I and Type II error.

Critical Effect Size	Choice of *α*	*α*	*β*	*ω*	Result^a^
large (*δ*≥1.5 *σ*_p_)	Standard	0.05	0.493	0.272	non-significant
	Optimal	0.191	0.212	0.202	significant
medium (*δ*≥*σ*_p_)	Standard	0.05	0.738	0.394	non-significant
	Optimal	0.266	0.372	0.319	significant
small (*δ*≥0.5 *σ*_p_)	Standard	0.05	0.898	0.474	non-significant
	Optimal	0.323	0.563	0.443	significant

a *p*-value used for significance testing is 0.14 [10].

Probabilities are calculated for a two-sample *t*-test (two-tailed) with n_1_ = 3, n_2_ = 8, and *σ*_p_ = 17.27, from [10].

### Case study 2: regression with high power

Now consider a study examining expression of a priori defined gene sets within human diabetic muscle tissue to determine whether there are sets of genes whose expression is correlated with insulin resistance and aerobic capacity [11]. One of the gene sets examined was a co-regulated set of genes involved in oxidative phosphorylation (OXPHOS-CR), and the authors wanted to test whether there was a relationship between the mean expression of this gene set in the muscle tissue of individuals and the total-body aerobic capacity of individuals (VO2max). The authors tested the hypotheses *H*_O_: there is no relationship between mean expression of OXPHOS-CR genes and VO2max of individuals, and *H*_A_: there is a relationship between mean expression of OXPHOS-CR genes and VO2max of individuals. The mean expression of OXPHOS-CR genes and the VO2max was measured in 43 age-matched male individuals with different levels of glucose tolerance (i.e. *N* = 43). The authors performed a simple linear regression analysis on the data, using the clinical variable (VO2max) as the dependent variable and mean gene expression as the predictor variable. They found that 22% of the variability in VO2max could be explained by mean OXPHOS-CR gene expression (R^2^_adj_ = 0.22), and the relationship had a *p*-value of 0.0012, which was considered significant by the authors, using *α* = 0.05.

Despite the small *p*-value (0.0012), the significance of the regression depends on the method used to choose *α* and the desired critical effect size (Table 2). As there was no justification for unequal prior probabilities of hypotheses or costs of Type I and Type II errors apparent in the introduction of the paper, we assumed equal prior probabilities and costs of errors. In the absence of a strong rationale for any particular critical effect size, we chose to calculate optimal *α* levels for 3 different potential critical effect sizes; an effect size we consider to be ‘large’ (R^2^≥0.75), where the independent variable explains 75% of the variability in the dependent variable (representing a strong relationship between the dependent and independent variables), and 2 smaller effect sizes (R^2^≥0.5 and R^2^≥0.25) where 50% and 25% of the variability in the dependent variable could be explained by the variability in the independent variable (representing weaker relationships between the dependent and independent variables). Using the traditional *α* of 0.05, the averages of Type I and Type II error at the critical effect sizes were 0.0250, 0.0251, and 0.0568, for the large, medium and small effect sizes, respectively. Using the optimal *α* approach, the averages of Type I and Type II errors were smaller, (0.0000383, 0.00381 and 0.0567, for the large, medium and small effect sizes, respectively) and had optimal *α* levels of 0.0000286, 0.00378 and 0.0531 and for the large, medium and small critical effect sizes, respectively. The conclusions reached using the optimal *α* approach are consistent with the conclusions reached by the authors using *α* = 0.05, except in the case of the large critical effect size, which represents a situation where the authors would only be interested in detecting a strong relationship between the dependent and independent variables. If detecting only strong relationships between variables is important then for 43 samples, *α* can be set at a very low level and there will still be very high statistical power to detect a strong relationship. The use of *α* = 0.05 for this test in cases where only strong relationships would be important to detect if they exist results in an unnecessarily high level of Type II error and keeps the average of *α* and *β* unnecessarily high - holding *α* at 0.05 makes 0.025 the smallest possible average of *α* and beta, when beta approaches 0. If relationships as weak as R^2^ = 0.25 are important to be able to detect for this test, then the optimal *α* level is close to *α* = 0.05, and results in both methods concluding that the observed relationship of R^2^ = 0.22 is significantly different from no relationship. It would be unusual to set a critical effect size at a value as large as R^2^ = 0.75 without a very strong rationale and so optimal *α* would have lead to similar conclusions as using traditional *α* = 0.05 in most cases.

**Table 2:** Probabilities of Type I (*α*), Type II (*β*) and average error (*ω*), with corresponding test conclusions for large, medium and small effect sizes (*δ*) using standard *α* levels and by setting *α* to minimize combined probabilities of Type I and Type II error.

Critical Effect Size	Choice of *α*	*α*	*β*	*ω*	Result^a^
large (R^2^≥0.75)	Standard	0.05	7.37*10^−11^	0.0250	significant
	Optimal	0.0000286	0.0000266	0.0000276	non-significant
medium (R^2^≥0.5)	Standard	0.05	0.000136	0.0251	significant
	Optimal	0.00378	0.00384	0.00381	significant
small (R^2^≥0.25)	Standard	0.05	0.0635	0.0568	significant
	Optimal	0.0531	0.0603	0.0567	significant

a *p*-value used for significance testing is 0.0012 [11].

Probabilities are calculated for a simple linear regression with *N* = 43, from [11].

### Case study 3: analysis of variance with unequal cost ratios

Third, consider a study examining differences in crop yields among three soil types, sand, clay and loam [12]. The authors tested the hypotheses *H*o: there are no differences in mean crop yield among the three soil types (*μ*1 = *μ*2 = *μ*3), and *H*a: there are differences in mean crop yield among the three soil types (*μ*1≠*μ*2≠*μ*3). Crop yields were measured in 10 randomly selected fields for each of the 3 soil types (i.e. *N* = 30, *k* = 3). The mean observed crop yields were 9.9, 11.5 and 14.3 units for sand, clay and loam, respectively, and the observed within-group pooled standard deviation was 3.4. The researchers performed a one-way ANOVA on these data, and reported a *p*-value of 0.02495, which would be considered significant at *α* = 0.05.

The significance of these data depend on the method used to choose *α*, the critical effect size and relative seriousness of Type I vs. Type II errors (Table 3). For simplicity, we have assumed that the researchers would consider it important to be able to detect a standard deviation among group means that is at least as large as the standard deviation within-groups (i.e. critical effect size of SD_among groups_/SD_within groups_ = 1). We assumed equal prior probabilities of null and alternate hypotheses and examined the influence of 3 different Type I/Type II error cost ratios, a situation where the cost of a Type I error is 4 times as serious as the cost of a Type II error; a situation where the relative cost of Type I and Type II error are equal, and a situation where the cost of a Type I error is 1/4 the cost of a Type II error. Using the traditional *α* of 0.05, *β* and the average of the probabilities of Type I and Type II error remain constant for the different Type I/Type II error cost ratios. This is not ideal because there is no logical rationale for maintaining the same willingness to make a Type I error as the relative costs of Type I and II errors change. However, using optimal *α* results in *α*, *β* and the cost-weighted average of the probabilities of Type I and Type II error changing under different cost ratio scenarios. For the critical effect size we assumed in this example, the optimal *α* results in the test being considered non-significant (contrary to the outcome using *α* = 0.05) except for the case where the cost of Type I error is considered to be one quarter the cost of Type II error. When costs of Type I and Type II error are considered equal in order to minimize the average of both Type I and Type II error, the optimal *α* is 0.0142, making the optimal conclusion ‘non-significant’ for this critical effect, with 98.8% power. For each scenario, the conclusion reached using an optimal *α* was most appropriate for the data, given the differing relative costs of Type I and Type II error.

**Table 3:** Probabilities of Type I (*α*), Type II (*β*) cost-weighted average error (*ω*_c_), and average error (*ω*), with corresponding test conclusions for Type I/Type II error cost ratios of 4, 1, and 0.25 using standard *α* levels and by setting *α* to minimize cost-weighted average of probabilities of Type I and Type II error.

Type I/Type II error cost ratio	Choice of *α*	*α*	*β*	*ω*_c_	*ω*	Result^a^
4	Standard	0.05	0.00213	0.0404	0.0261	significant
	Optimal	0.00658	0.0274	0.0107	0.0170	non-significant
1	Standard	0.05	0.00213	0.0261	0.0261	significant
	Optimal	0.0142	0.0121	0.0132	0.0132	non-significant
0.25	Standard	0.05	0.00213	0.0117	0.0261	significant
	Optimal	0.0282	0.00506	0.00967	0.0166	significant

a *p*-value used for significance testing is 0.02495 [12].

Probabilities are calculated for a one-way ANOVA with *N* = 30, *k* = 3, and *σ*_p (within groups)_ = 3.4, and critical effect size = *σ*_p (within groups)_ from [12].

### Conclusion

There is a growing consensus that *α* = 0.05 is not an ideal method for statistical decision-making, and we have developed an approach to setting *α* that improves our ability to reach appropriate conclusions. Setting an optimal *α* does require the careful consideration of critical effect size, costs of Type I and II error and prior probabilities but these characteristics of any study always warrant consideration. Setting *α* at any particular value (including using *α* = 0.05) implies specific critical effect sizes and relative costs of Type I and II errors. The only difference between using an optimal *α* or a standard *α* is whether critical effect sizes and relative costs are thoughtfully considered and stated, or implied and unstated. The standard *α* approach has, therefore, not served science well; resulting in mistaken conclusions more often than necessary and allowing scientists to have the standard *α* level arbitrarily determine the size of effect that they are likely to consider significant, if the effect is real, and to have this arbitrary *α* level determine the relative chance of Type I vs. Type II error for meaningful potential effect sizes. We have described a rigorous approach to setting *α* that addresses both of these important (albeit, difficult) decisions and has an explicit and defensible objective, minimizing the combined probabilities or costs of making errors, and we recommend that this approach be applied in any field where null hypothesis significance tests are being used. More research is needed concerning how to estimate and properly incorporate appropriate critical effect sizes and relative costs of Type I and II errors into decision-making thresholds for pure and applied research questions. We are optimistic that the use of the optimal *α* approach will spur research in these areas and hope that these research gaps do not prevent the implementation of a technique that, even in its most basic form, offers strong improvements in probability of errors and decision-making transparency, over the arbitrary standard *α* approach. We acknowledge that the optimal *α* approach to null hypothesis significance testing is not without drawbacks and shortcomings, the traditional approach of setting *α* = 0.05 has these same drawbacks and shortcomings, but does a much worse job of addressing them. When the weaknesses of our approach are weighed against the weaknesses of the traditional *α* = 0.05 approach, it is impossible to ignore that our approach is a dramatic improvement, even if it is not perfect. Our goal is to improve upon an obviously flawed hypothesis-testing system while working under the constraints of that system, because we acknowledge that researchers are not very likely to abandon an approach that is so easy-to-use and so widely understood.

## Analysis

### How to calculate the optimal α (post data collection, pre-NHST)

#### 1. Determine critical effect size

This must be determined *a priori*, and is the magnitude of the effect that would be considered important to detect, if real. The type of effect size will be dependent on the type of NHST to be conducted. We suggest a value that is meaningful for the study, and is based on knowledge of the system or what other studies have observed to be important in your system. For more information on choosing and calculating a critical effect size, we recommend the reviews [14], . If you choose an absolute (i.e. unstandardized) critical effect size, a measure of variability must also be provided.

#### 2. Choose whether to minimize combined probabilities of Type I and Type II error (ω) or relative cost of errors (ω_c_)

If the average of the probabilities of Type I and Type II error is to be minimized, then the relative costs of Type I and Type II errors can be ignored and Equation 1 should be used. If cost of errors is to be minimized, then an estimate of the Type I/Type II error cost ratio is needed and Equation 2 should be used. Equations can also be weighted by the prior probabilities of null and alternate hypotheses at this stage, for special cases where prior probabilities are known with some degree of confidence.

#### 3. Calculate optimal α

Using the chosen equation of *ω* (average error) from step 2, calculate *ω* for a range of *α* levels. For each *α* level chosen, the associated *β* is 1 minus statistical power, calculated using the chosen *α* level, the study sample size and the critical effect size chosen in step 1. Look at the *ω* levels calculated for the range of *α* levels and find the value for *α* that results in the lowest resulting *ω*. This is the optimal *α* that minimizes the probability/cost of making a wrong conclusion. We have developed R code that will conduct this iterative process of determining the optimal *α* through examination of *ω* over a range of *α* levels (Text S1, Text S2, Text S3).

#### 4. Report all of the following values for each NHST

Sample size, chosen critical effect size(s), chosen relative cost of Type I to Type II error (if applicable), optimal *α*, optimal *β*, and *ω* (average of Type I and Type II error).

## Supporting Information

Text S1 **R code to calculate an optimal** ***α*** **for one-sample, two-sample, or paired** ***t*** **-tests for one or two tailed hypotheses is provided as supplementary information.** This will perform the iterative optimization steps for choosing an optimal *α* based on sample size(s) and Cohen's *d* critical effect size provided by the user.
(TXT)

Text S2 **R code to calculate an optimal** ***α*** **for two-tailed simple linear correlation and regression tests is provided as supplementary information.** This will perform the iterative optimization steps for choosing an optimal *α* based on sample size and correlation coefficient critical effect size provided by the user.
(TXT)

Text S3 **R code to calculate an optimal** ***α*** **for ANOVA is provided as supplementary information.** This will perform the iterative optimization steps for choosing an optimal *α* based on the numerator degrees of freedom, denominator degrees of freedom and Cohen's *f* ^2^ critical effect size provided by the user.
(TXT)
